# Effect of Acrylic Emulsion on Drying Shrinkage, Mechanical Properties, and Hydration Behavior of UHPC Matrix

**DOI:** 10.3390/gels12100926

**Published:** 2026-10-15

**Authors:** Jie Fan, Sijie Deng, Gengying Li, Yong Yu

**Affiliations:** 1 School of Future Transportation, Guangzhou Maritime University, Guangzhou 510725, China; jfan1988@163.com (J.F.);; 2 School of Civil Engineering and Transportation, Foshan University, Foshan 528200, China

**Keywords:** acrylic emulsion (AE), UHPC matrix, C-S-H gel, drying shrinkage, hydration behavior, mechanical properties

## Abstract

Significant drying shrinkage and early-age cracking have remained critical constraints to the broader application of ultra-high performance concrete (UHPC). This study attempted to address these limitations through the addition of acrylic emulsion (AE), and the influence of its content (0–10 wt.%) on the setting time, mechanical properties, drying shrinkage, hydration behavior, and microstructure of UHPC matrix was systematically investigated. The results show that AE addition delayed setting and early hydration, substantially reducing cumulative heat release and thereby lowering the risk of thermal cracking. AE incorporation reduced compressive strength, and this adverse effect became more pronounced at higher dosages. In contrast, the formation of intertwining polymer films within the hydration products provided effective pore-filling and “micro-fibre” effects, which enhanced flexural strength development and alleviated drying shrinkage that originates principally from moisture loss within the C-S-H gel. At an optimal dosage of 5 wt.%, AE reduced the 14-day and 90-day drying shrinkage of UHPC matrix by 28% and 25%, respectively, while increasing the 90-day flexural strength by approximately 3%. However, a further increase in the dosage deteriorated overall performance, primarily because excessive polymer promoted agglomeration and introduced more air bubbles. These findings provide a theoretical foundation for designing low-shrinkage, low-heat-release UHPC tailored for large-volume engineering structures.

## 1. Introduction

Ultra-high performance concrete (UHPC) achieves a highly compact microstructure through measures such as an extremely low water-to-binder ratio (typically ≤ 0.20), optimised grading of fine aggregates, the incorporation of highly reactive ultrafine mineral admixtures like silica fume, and the use of high-efficiency water reducers. As a result of this tailored compositional design, UHPC exhibits significantly superior mechanical properties compared to ordinary concrete, along with excellent impermeability, enhanced resistance to chemical erosion, and long-term durability [1,2,3,4]. These superior performance characteristics make UHPC particularly well-suited for structurally demanding applications, such as long-span bridges, high-rise buildings, offshore platforms, and protective military structures. Despite these advantages, the practical application of UHPC remains constrained by several notable drawbacks due to its extremely low water content and high cementitious material usage. In particular, the very low water-to-binder ratio leads to incomplete cement hydration, significant internal self-desiccation effects, and substantial shrinkage, which can exceed 1000 μm/m—far higher than that observed in ordinary concrete [5,6]. Under constrained conditions, such excessive early-age shrinkage readily promotes cracking, thereby compromising the structural integrity and durability. In addition, the hydration of the cementitious system in UHPC is accompanied by a rapid and localised release of heat, which results in a steep temperature rise. In mass concrete applications, the accumulated internal heat cannot be dissipated quickly, potentially leading to high thermal stress and a markedly increased risk of thermal cracking [7,8]. Therefore, effectively controlling the shrinkage behavior and reducing the hydration heat of UHPC are key challenges for enabling its broader engineering applications.

Fundamentally, drying shrinkage in cement-based materials, including shrinkage in general, is governed by the calcium silicate hydrate (C-S-H) gel, the principal binding phase of the hardened cementitious matrix. From the standpoint of pore-structure mechanics, the hardened matrix is a porous network of C-S-H gel permeated by capillary pores; as the ambient relative humidity falls, water held within the gel pores and the interlayer space is progressively lost, generating capillary tension and disjoining pressure that drives contraction of the gel skeleton [5,6,9]. To suppress shrinkage in UHPC and improve its dimensional stability, existing technical approaches primarily rely on the incorporation of expansion agents, shrinkage-reducing agents, and superabsorbent polymers (SAPs). Expansion agents, including MgO, CaO, and calcium sulphoaluminate, promote the formation of expansive products, such as ettringite, through specific chemical reactions during cement hydration, thereby partially compensating for shrinkage-induced stresses [9,10]. Nevertheless, the use of these agents may increase pore size and overall porosity, which could adversely affect long-term mechanical strength and durability [11]. In contrast, shrinkage-reducing agents function by lowering the surface tension of the pore solution, thus slowing the development of capillary stresses and improving the retention of internal relative humidity. This mechanism effectively restrains autogenous drying shrinkage [12,13]. It should be noted that their effectiveness in systems with low water-to-binder ratios is often limited [14]. As an elastic gel, SAP is capable of absorbing water at levels tens to hundreds of times greater than its own weight and subsequently releasing it. When used as an internal curing material in UHPC, SAP absorbs a significant amount of water during the mixing stage and slowly releases it during the hydration process, thereby mitigating drying and enhancing the degree of hydration [15,16]. However, the residual pores formed after SAP releases water often reduce the strength and density of the matrix [17,18]. Additionally, premature water uptake by SAP during mixing can lead to rapid water loss, negatively affecting the fresh-state workability of UHPC [19].

In recent years, polymer emulsions have emerged as highly effective modifiers for cement-based materials, offering distinct advantages over conventional approaches. After incorporation into the cement matrix, polymers form a continuous film network that bridges microcracks and redistributes applied stresses, thereby significantly enhancing flexural strength and overall toughness [20,21,22,23]. Moreover, the filling effect provided by polymer emulsions in capillary pores effectively blocks the penetration pathways of deleterious agents such as moisture and chloride ions, which substantially enhances the durability of cement-based materials and extends the service life of structures exposed to harsh environments [24,25,26,27]. Beyond these mechanical and durability benefits, polymer emulsions also exhibit a certain degree of water retention and internal curing. By retaining moisture within the capillary and gel pores and slowing its loss from the C-S-H gel, they reduce drying shrinkage caused by rapid water evaporation and suppress the development of autogenous shrinkage, consequently lowering the risk of early cracking [28,29]. Yang et al. [30] showed that adding 20 wt.% carboxylated styrene−butadiene emulsion markedly improved the flexibility and flexural strength of cement-based materials, while simultaneously reducing the 28-day drying shrinkage by approximately 10%. In a related study, Ding et al. [31] indicated that the addition of a styrene−acrylic emulsion did not enhance the flexural and compressive strength of calcium sulphoaluminate (CSA) cement paste; however, it effectively reduced drying shrinkage and resulted in a pronounced increase in the ratio of flexural to compressive strength. Furthermore, Li et al. [32] demonstrated that the use of styrene−butadiene rubber significantly improved the resistance of CSA cement paste to carbonation and sulphate attack, while also reducing drying shrinkage and capillary water absorption.

Previous studies on the application of polymer emulsions have primarily focused on ordinary concrete systems characterised by relatively high water-to-binder ratios and low mechanical strength. Hence, the impact of polymer emulsions on the performance of UHPC remains unclear, and the underlying mechanisms are yet to be elucidated. This knowledge gap may limit the widespread application of polymer emulsions for enhancing the properties of UHPC, thus necessitating further research. It should be noted that, while UHPC is commonly used as a fibre-reinforced composite in structural engineering, the fibres mainly control post-cracking tensile resistance and toughness, and can introduce additional bridging, confinement, and interfacial effects. In addition, the presence of fibres may obscure the intrinsic matrix-level modification effect of the polymer emulsion, making it difficult to evaluate. To isolate the intrinsic matrix-level modification mechanism of the polymer emulsion, this study focuses on the fibre-free matrix phase of UHPC. In the literature [33,34,35,36], this fibre-free matrix phase is widely and specifically designated as the “UHPC matrix”, distinguishing it from fibre-reinforced UHPC. This UHPC matrix consists of cement, supplementary cementitious materials, and quartz sand. For brevity, this fibre-free system is referred to as the UHPC matrix hereafter. In this study, an acrylic emulsion (AE) was employed to modify the UHPC matrix, and the effects of its incorporation on setting time, mechanical properties, drying shrinkage, and capillary water absorption were systematically investigated. In addition, the mechanisms governing the observed macroscopic responses were further analysed through hydration heat monitoring, thermogravimetric (TG) analysis, and scanning electron microscopy (SEM) observations.

## 2. Results and Discussion

### 2.1. Setting Time

**Figure 1:**
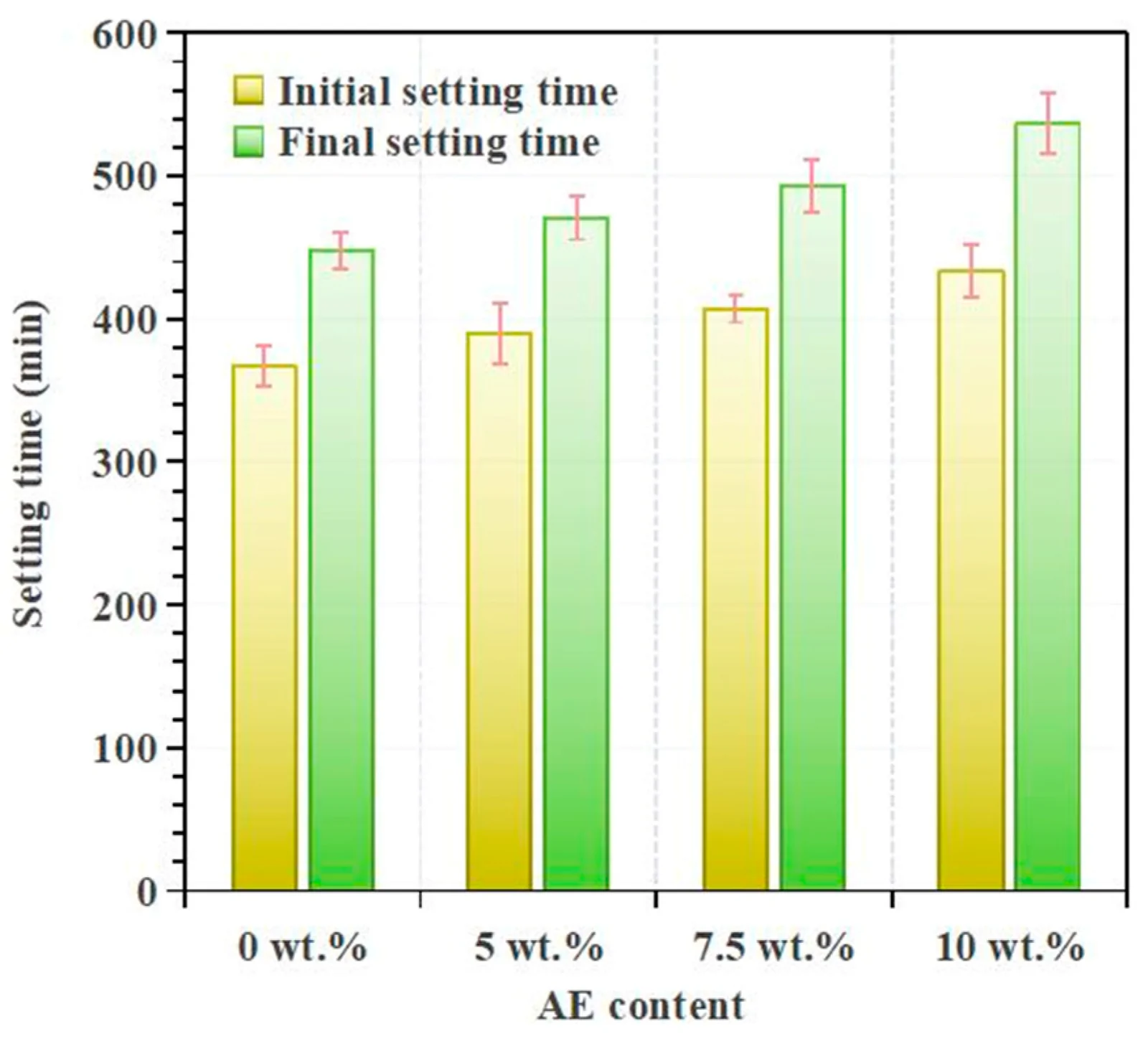
Effect of AE on the setting time of UHPC matrix.

The test results of setting time for fresh UHPC matrix with varying AE contents are shown in Figure 1. In the absence of polymer, the measured initial and final setting times were 367 min and 448 min, respectively. As the AE content increased, a clear delay in setting was observed, with this retarding effect becoming more pronounced at higher addition levels. When the AE content reached 10 wt.%, the initial and final setting times were extended to 434 min and 537 min, respectively. The mechanism of AE delaying the setting time of fresh UHPC matrix can be attributed to two primary aspects. First, AE molecules adsorb onto and encapsulate cementitious materials, thus hindering direct contact between unhydrated mineral components and water molecules [37]. Second, the abundant -COO^−^ groups on the AE surface readily complex with Ca^2+^ ions, prolonging the induction period of hydration [38]. This interaction further slows down the polymerisation reaction rate of the UHPC matrix system, resulting in an extended setting process.

### 2.2. Hydration Heat

**Figure 2:**
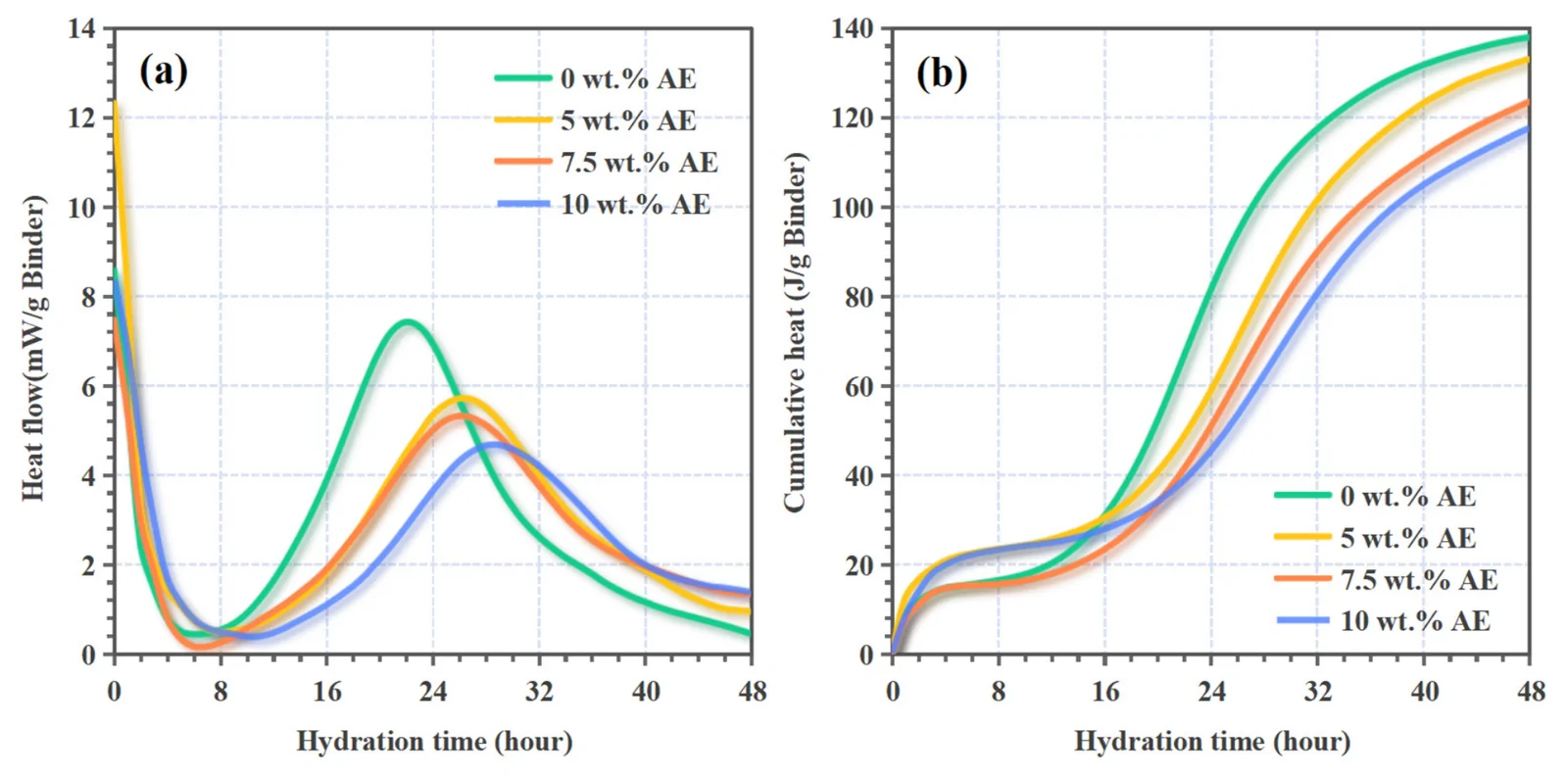
Heat release rate of hydration (**a**) and cumulative heat release (**b**) for UHPC paste with different AE contents.

Figure 2 shows the effect of AE addition on the hydration behavior of the UHPC paste. From the evolution of the heat-flow curves, the hydration of all mixtures could be clearly divided into four successive stages, namely the initial, induction, acceleration, and deceleration stages. The initial stage occurred shortly after the contact between the cementitious materials and mixing water, characterised by a short-lived and sharp exothermic peak. This period was followed by a prolonged interval of very low heat release lasting several hours, which is characteristic of the induction stage. Upon entering the acceleration stage, the hydration exothermic rate increased markedly, accompanied by a pronounced rise in cumulative heat release. The acceleration stage has commonly been associated with the hydration of tricalcium silicate in previous reports [39,40]. As shown in Figure 2a, all investigated samples exhibited a single major exothermic peak located at the transition from the acceleration stage to the subsequent deceleration stage. The incorporation of AE significantly delayed the onset of the acceleration period, and this retarding effect became more pronounced with increasing AE dosages. Compared to the control group without AE (0 wt.% AE), the UHPC paste containing 10 wt.% AE exhibited an approximately 7 h shift in the main exothermic peak. This retardation effect can be primarily attributed to two main reasons. Firstly, the adsorption of AE molecule onto cement particle surfaces generates a film-like coating that hinders the effective diffusion of key ions such as Ca^2+^ and SiO_4_^2−^, thereby slowing down the kinetics of the hydration process. Secondly, the abundant -COO^−^ groups on the surface of AE molecule provide sufficient sites to capture adjacent Ca^2+^ ions, which lowers the concentration of free Ca^2+^ ions in the aqueous phase and consequently inhibits the nucleation and growth of hydration products [41]. The cumulative heat-release profiles of UHPC pastes with different AE contents (Figure 2b) further confirmed the trends discussed earlier. Compared with the control sample, the presence of AE resulted in a clear decrease in the cumulative heat release within 48 h, and the magnitude of this reduction increased progressively with higher AE content. Specifically, the UHPC paste containing 10 wt.% AE exhibited a cumulative heat release of only 116 J/g within 48 h, representing a 16% decrease relative to the control sample (138 J/g). Notably, the delayed early-age hydration caused by AE addition was beneficial in mitigating localised stress concentrations induced by the rapid initial heat release of UHPC paste, thereby reducing the risk of microcrack formation.

### 2.3. Flexural and Compressive Strength

**Figure 3:**
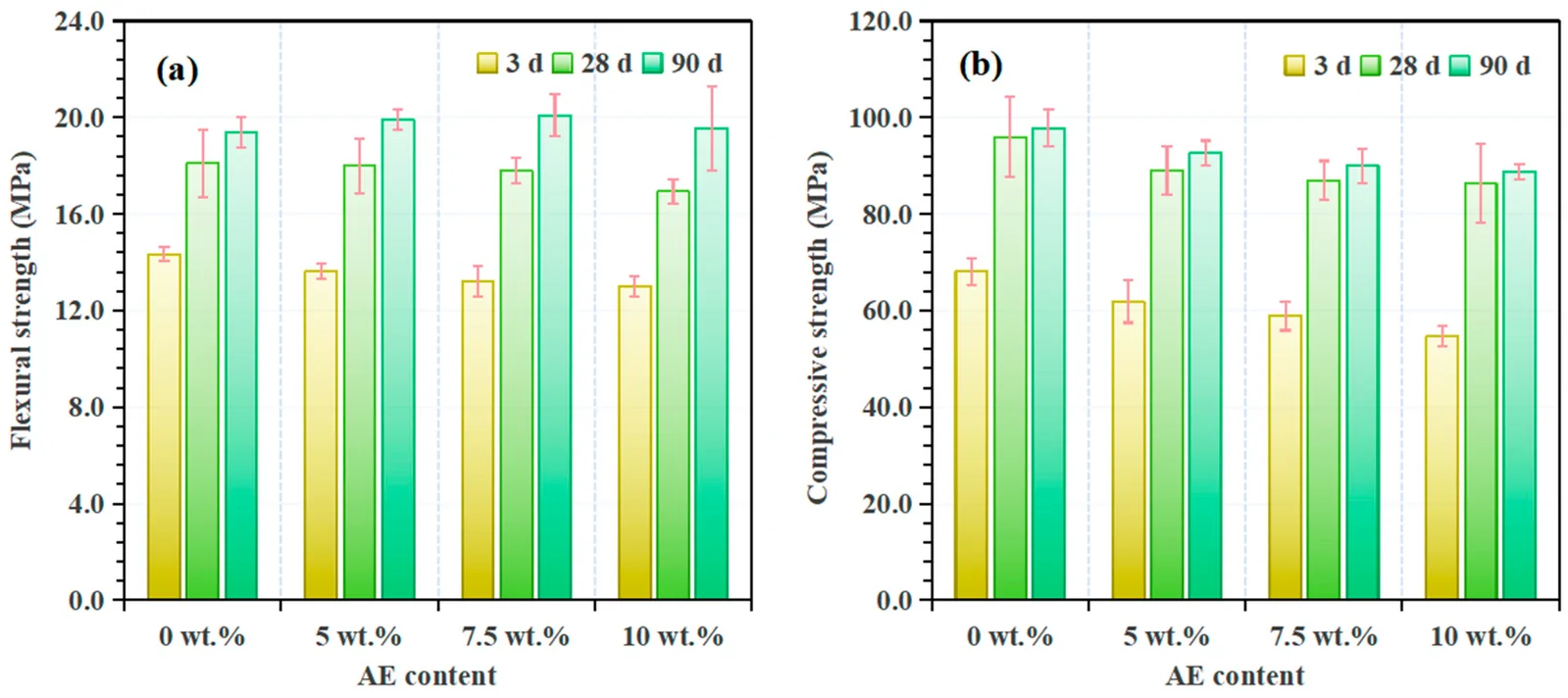
Effect of AE on flexural (**a**) and compressive strength (**b**) of UHPC matrix.

Figure 3a depicts the flexural strength of UHPC matrix with different AE dosages at curing ages of 3, 28, and 90 days. A continuous increase in flexural strength with curing time was observed for all samples. At the curing age of 3 days, the control sample without AE exhibited the highest early-age flexural strength, reaching 14.3 MPa. In contrast, a progressive reduction in early flexural strength was noted with increasing AE content. Specifically, the 3-day flexural strength of the UHPC matrix with AE dosages of 5 wt.%, 7.5 wt.%, and 10 wt.% decreased by 5%, 8%, and 9%, respectively, compared to the control. This loss in early-age strength is ascribed to the adsorption of polymer on the surfaces of unhydrated cement particles, which blocks active nucleation sites and hinders hydration. The continued hydration reaction led to a gradual reduction in the moisture content within the matrix, forming a more extensive organic–inorganic network structure where polymer films became interlaced with the hydration products. This network acted as “micro-fibres,” enhancing the toughness of the composite and inhibiting crack initiation and propagation [42,43]. Additionally, the polymer films partially filled capillary pores within the matrix, enabling absorption and reflection of the external force applied during fracture [44,45]. At 28 days, the flexural strengths of UHPC samples containing 5 wt.% and 7.5 wt.% AE were comparable to that of the control mixture. After 90 days of curing, the flexural strength increased by approximately 3% and 4% for the UHPC matrices containing 5 wt.% and 7.5 wt.% AE, respectively, compared with the control, whereas the 10 wt.% AE mixture showed no significant improvement. These results indicate that an appropriate amount of AE addition contributes to improving the flexural strength of UHPC matrix at the intermediate and later curing stages.

Figure 3b shows the compressive strength of UHPC matrix with varying doses of AE at curing ages of 3, 28, and 90 days. A clear and monotonic reduction in compressive strength was observed with increasing AE content at all curing ages, and the loss was more significant at early ages. In particular, the mixture containing 10 wt.% AE showed reductions of 19%, 10%, and 9% at 3, 28, and 90 days, respectively, compared with the control sample without AE. For the mixtures containing 5 wt.% and 7.5 wt.% AE, the corresponding reductions were approximately 5–9% and 8–14%, respectively, across the tested curing ages. Among the AE-modified mixtures, the 5 wt.% AE mixture exhibited the smallest reduction in compressive strength. The deterioration in the mechanical performance of UHPC matrix induced by AE can mainly be attributed to two fundamental factors. Firstly, due to the much lower elastic modulus of the polymer compared to UHPC matrix, the presence of AE within the hydration product network weakens the transmission of compressive stress and leads to the formation of structural defects [46,47]. Secondly, the adsorption effect of the polymer and the binding of its surface -COO− groups with Ca^2+^ delay the nucleation and growth of hydration products and reduce the degree of hydration, thus impairing the development of compressive strength, especially at early stages [48]. Owing to these combined effects, the incorporation of AE additives results in significantly different responses of compressive strength and flexural strength in UHPC matrix.

### 2.4. Drying Shrinkage

**Figure 4:**
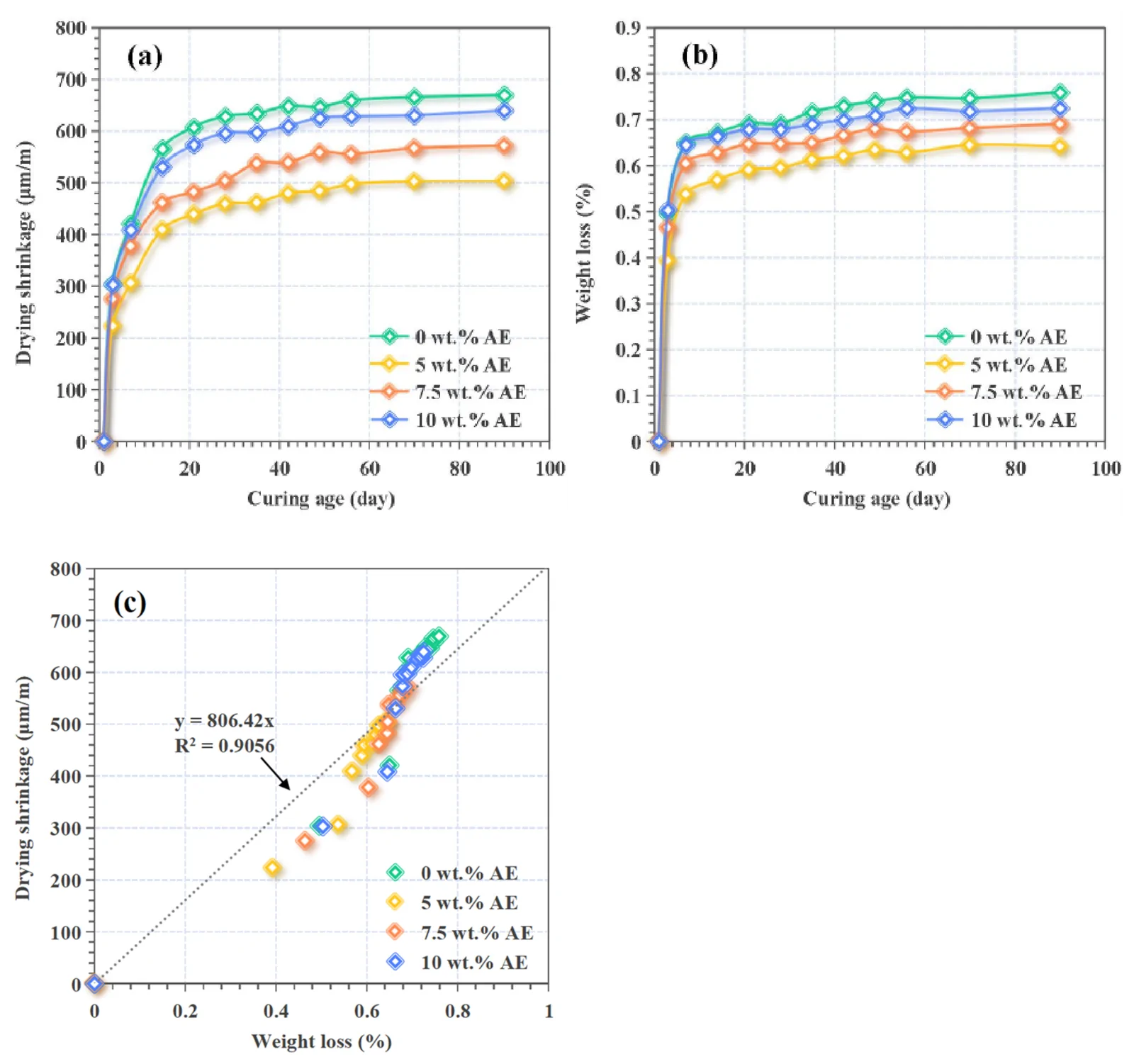
Drying shrinkage (**a**), weight loss (**b**) and their relationship (**c**) of UHPC matrix with different AE contents.

The effect of AE addition on the volumetric stability of UHPC matrix was investigated through drying-shrinkage experiments. Figure 4a shows the shrinkage rates of each group at different curing ages. It was observed that the shrinkage rates of all samples continuously increased with age, showing a rapid growth trend within the first 14 days, followed by a slower growth phase between 14 and 56 days, and a gradual stabilisation after 56 days. The control sample exhibited a drying shrinkage of 669.6 × 10^−6^ at the curing age of 90 days, which was approximately 1.18 times that at 14 days. The addition of AE evidently improved the volume stability of the UHPC matrix. At the same curing age, the sample containing 5 wt.% AE consistently displayed the lowest drying shrinkage. Specifically, the shrinkage at 14 and 90 days were 409.0 × 10^−6^ and 503.4 × 10^−6^, representing reductions of 28% and 25%, respectively, compared to the control sample. When the AE content was further increased to 7.5 wt.% and 10.0 wt.%, a significant increase in drying shrinkage was observed. Nevertheless, the shrinkage values for these mixtures remained lower than those of the control sample at all curing ages. These results indicate that the introduction of AE at an appropriate dosage is an effective approach for mitigating drying shrinkage in UHPC matrix, particularly at early stages. Under identical curing conditions, the weight loss of AE-modified UHPC matrix was determined at different ages, and the corresponding data are presented in Figure 4b. For all samples, weight loss progressively increased with curing age and developed most rapidly during the early stage. A high linear correlation between drying shrinkage and weight loss was observed for all UHPC matrix specimens with different AE contents at different curing ages, and this correlation is represented by the line in Figure 4c. The UHPC matrix containing 5 wt.% AE exhibited the lowest weight loss at all curing ages, showing an approximate 16% reduction compared to the control sample at 90 days. Mechanistically, drying shrinkage in cement-based materials is governed by the calcium silicate hydrate (C-S-H) gel, the principal binding phase of the hardened matrix. As the ambient relative humidity falls, water held within the nano-sized gel pores and the interlayer space of the C-S-H gel is progressively depleted, and the resulting increase in capillary tension within the gel skeleton drives macroscopic contraction [49,50]. The incorporation of AE mitigates such shrinkage through two coupled effects. On the one hand, the AE forms a continuous polymer film during curing that seals capillary pores and, owing to the hydrophilic nature of the polymer, retains moisture within the gel pores and slows its loss from the C-S-H gel, thereby alleviating the capillary tension that drives gel-skeleton contraction [28,29] and reducing the evaporation of free water. On the other hand, AE also suppresses the early-stage hydration heat release, lowering the overall moisture demand and the extent of self-desiccation. Moreover, the interweaving arrangement of the AE film within the hydration products is expected to create a “micro-fiber effect,” which further restricts the development of drying shrinkage [51]. However, it should be noted that excessive AE dosage may induce particle agglomeration, leading to additional structural defects that negatively affect the microstructure of the composite material. This phenomenon is described in detail in the subsequent sections.

### 2.5. Capillary Water Absorption

**Figure 5:**
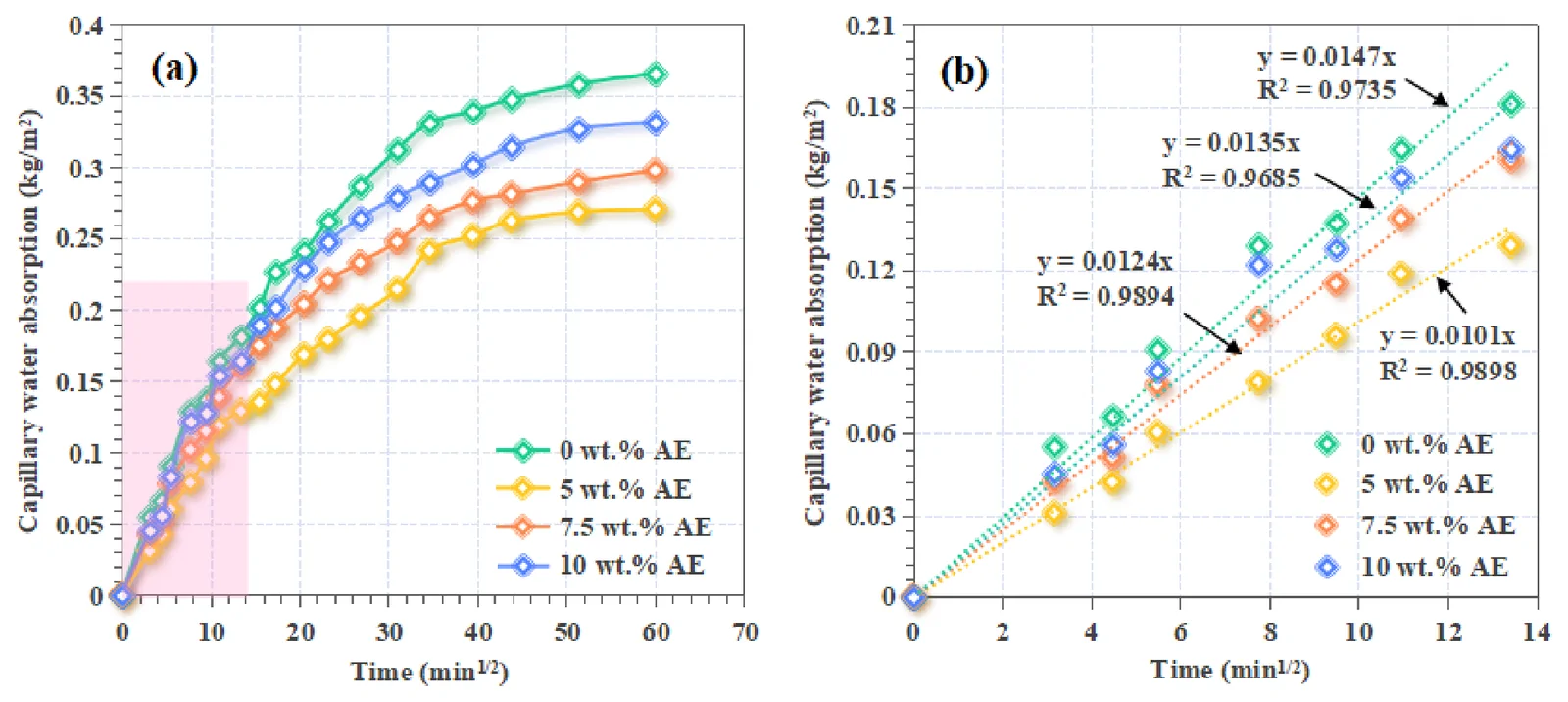
Capillary water absorption (**a**) and water absorption coefficient (**b**) of UHPC matrix with different AE contents.

Figure 5 displays the capillary water absorption curves and the water absorption coefficients for UHPC matrices with different AE contents. The capillary water absorption of all specimens showed a two-stage behavior, consisting of an initial rapid absorption stage within the initial 20 h, followed by a saturation stage. Among the four sets of samples, the UHPC matrix containing 5 wt.% AE exhibited the lowest cumulative capillary water absorption of 0.268 kg/m^2^ after 60 h of capillary absorption, which corresponded to a reduction of approximately 27% relative to the control group. In contrast, increasing the AE content to 7.5 wt.% and 10 wt.% resulted in smaller improvements, with the 60 h cumulative capillary water absorption decreasing by about 19% and 10%, respectively. Following the procedure described in [52], the slope of the rapid absorption stage of the capillary water absorption curve, in which the cumulative water absorption increases approximately linearly with the square root of time, was taken as the capillary water absorption coefficient to characterize the water penetration resistance. Figure 5b presents the linear fitting results for this stage; the fitting was performed using the data in the pink region of Figure 5a, and the slope of the fitted line corresponds to the capillary water absorption coefficient (kg·m^−2^·min^−0.5^). It was found that the addition of AE efficiently reduced the capillary water absorption coefficient, with the optimum performance achieved at an AE content of 5 wt.%. This improvement can be ascribed to the formation of a continuous polymer film within the UHPC matrix, which provides both pore-sealing and hydrophobic effects, thus effectively hindering the diffusion and transport of water molecules [53,54]. It is well known that improving the water resistance of cementitious materials can generally mitigate deterioration caused by aggressive media, such as acidic solutions and water-soluble salts [55], as well as damage induced by freeze–thaw cycles [56]. Therefore, the addition of an appropriate amount of AE is expected to impart UHPC matrix with better durability.

### 2.6. TG Analysis

**Figure 6:**
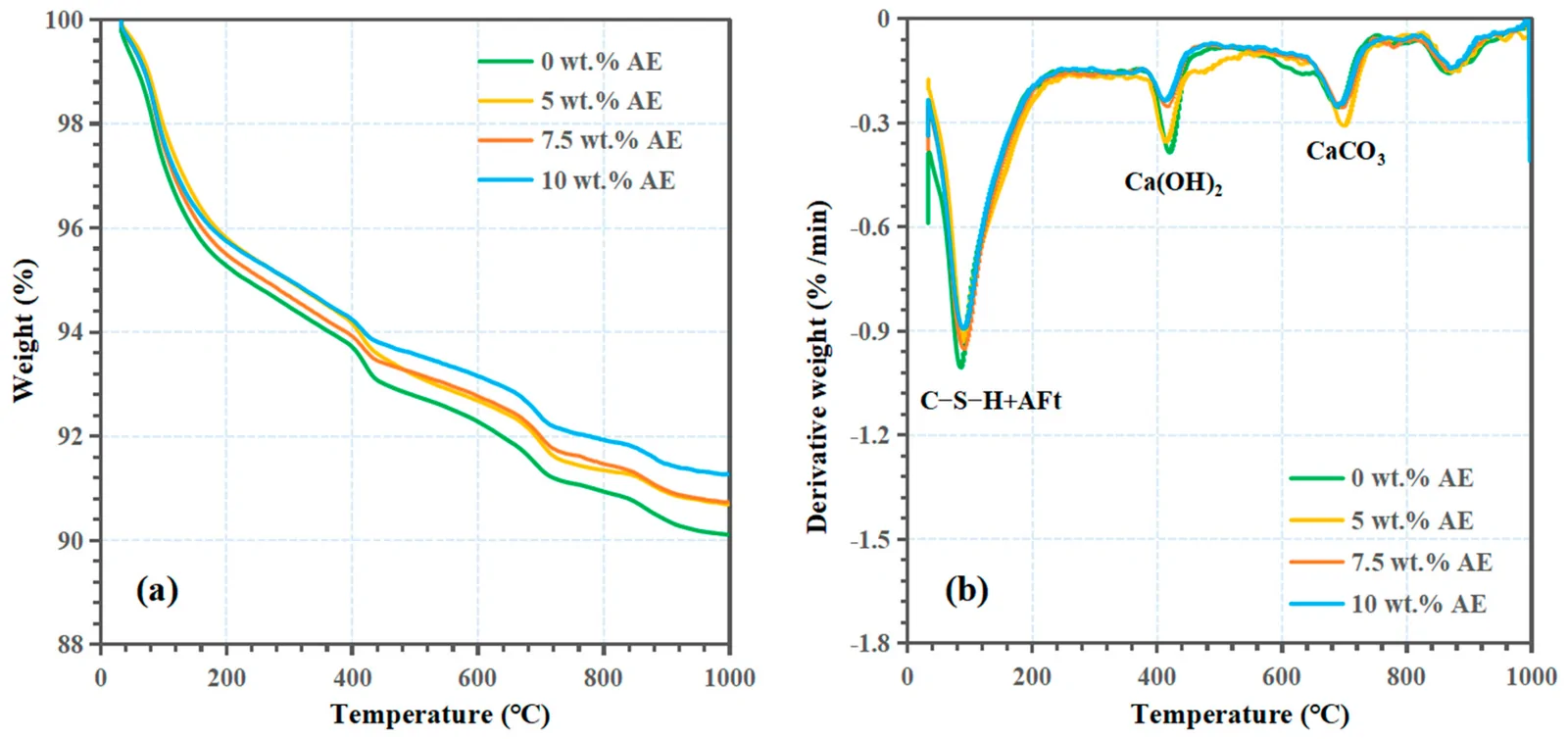
TG (**a**) and DTG (**b**) curves of UHPC matrix with different AE contents.

Figure 6 shows the TG and derivative thermogravimetric (DTG) curves of UHPC matrices with different AE contents after 28 days of curing. For all mixtures, thermal decomposition proceeded through three distinct stages of mass loss. The first stage, extending from approximately 40 to 200 °C, was associated with the release of free water and the dehydration of hydration products such as C-S-H gel and AFt [57]. Since all specimens were subjected to low-temperature drying for up to 7 days, the mass loss observed within this temperature range can be mainly attributed to the dehydration of C-S-H gel and AFt rather than to moisture evaporation. The second stage took place between around 350 °C and 450 °C, primarily due to the decomposition of calcium hydroxide. A third decomposition stage was observed in the range of about 620–750 °C, which is mainly related to the decarbonation of calcium carbonate accompanied by the release of CO_2_ [58]. Meanwhile, comparison of the DTG curves indicated that the addition of AE led to a noticeable reduction in the intensity of the mass-loss peak within the 40–200 °C range. This behavior may be related to the formation of the polymer film that encapsulates the cementitious particles, which could inhibit cement hydration as well as the secondary hydration reactions of SF and FAM. Moreover, for UHPC matrix containing AE, the mass-loss peak corresponding to calcium hydroxide showed some weakening, and this phenomenon became increasingly evident with higher AE content. This trend may be related to the complexation between the COO^−^ groups on the AE surface and Ca^2+^ ions [59]. Additionally, the use of AE resulted in a slight decrease in the decomposition temperatures of calcium hydroxide, as indicated by the leftward shift in the corresponding mass loss peaks. This behavior suggests an increase in the crystal structure defects of calcium hydroxide [60]. However, it should be noted that the polymer itself may also decompose, which could partially contribute to the observed mass loss; therefore, the TG/DTG results are used only for qualitative analysis of the changes in hydration products. Because the polymer decomposition would tend to increase, rather than decrease, the measured mass loss, the comparison of the total mass loss at 1000 °C remains valid and, indeed, tends to be conservative: the control group exhibited the largest overall mass loss, while the UHPC matrices containing AE showed lower mass losses. This trend suggests that AE addition reduces the overall mass loss, which may partly result from the inhibition of hydration product formation.

### 2.7. Microstructure Morphology Analysis

**Figure 7:**
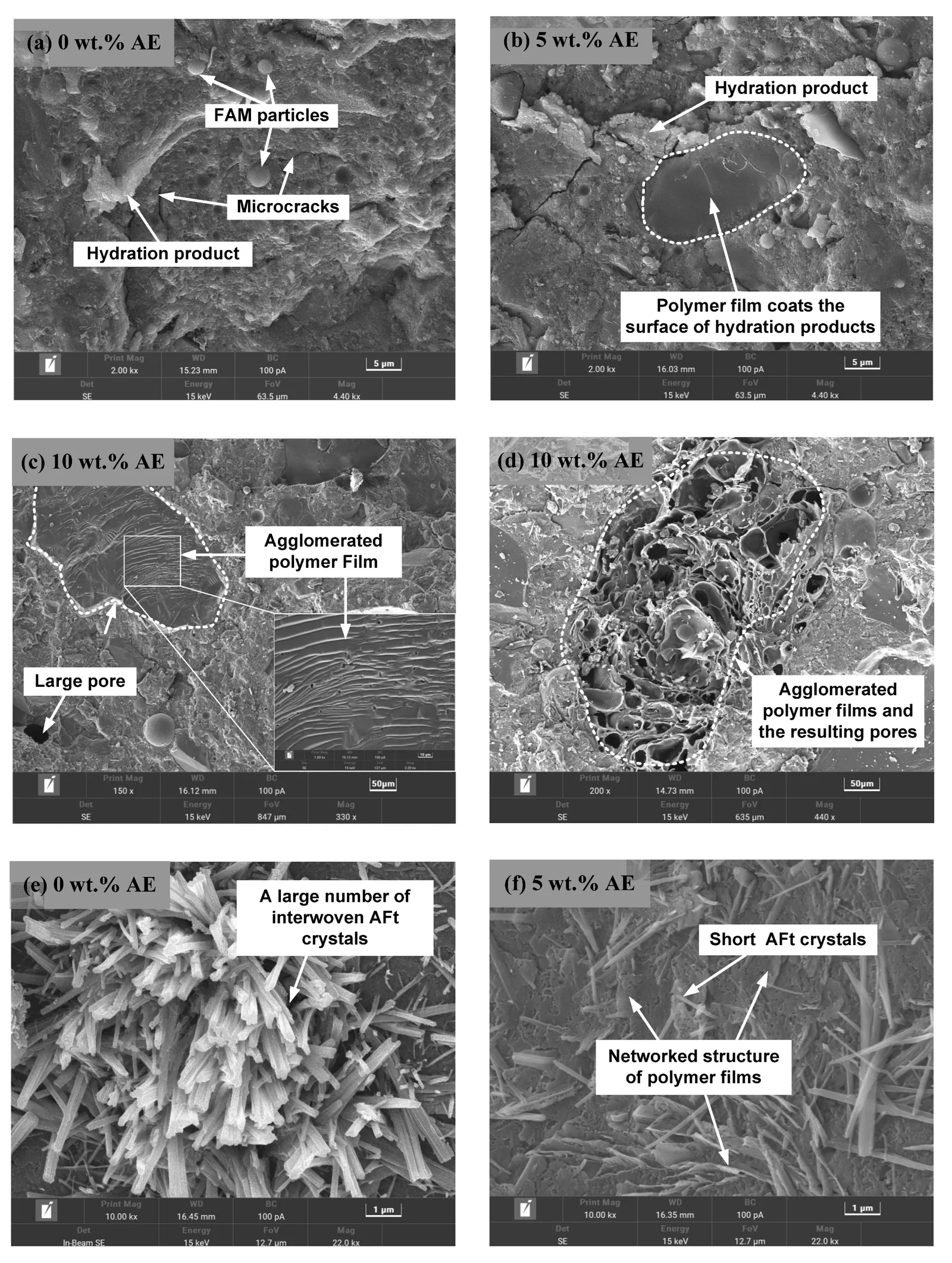
Typical SEM images of UHPC matrix with different AE contents: (**a**) 0 wt.% AE; (**b**) 5 wt.% AE; (**c**) 10 wt.% AE showing polymer film agglomerates on the matrix surface; (**d**) 10 wt.% AE showing polymer agglomerates between hydration products; (**e**) AFt morphology in the 0 wt.% AE sample; (**f**) AFt morphology and polymer films in the 5 wt.% AE sample.

To understand the effect of AE addition on the microstructural characteristics of UHPC matrix, specimens cured for 28 days were observed via SEM, and the related images are shown in Figure 7. As depicted in Figure 7a, the control sample without AE displayed a relatively dense and well-developed microstructure, where hydration products and partially hydrated FAM particles were clearly visible. On the contrary, in the UHPC matrix containing 5 wt.% AE (Figure 7b), in addition to hydration products and residual unhydrated particles, locally distributed polymer films covering the matrix surface were observed. Although these polymer films did not provide any bridging effect, they effectively prevented moisture loss from the matrix and hindered the intrusion of external solutions [32]. Consequently, the improved resistance of AE-containing UHPC matrix to drying shrinkage and capillary water absorption mainly stemmed from the formation of this characteristic microstructure. Figure 7c,d show representative micro-morphological features of the sample with 10 wt.% AE. The micrographs revealed that the addition of a high dosage of AE led to the formation of agglomerated polymer films. These agglomerates not only formed and covered the surface of the matrix (Figure 7c) but also appeared between hydration products (Figure 7d). The formation of large and continuous polymer film agglomerates not only impeded the normal hydration process but also introduced numerous structural defects within the matrix, which may explain the decline in mechanical performance and water resistance of samples with high AE content. In addition, the 10 wt.% AE sample exhibited a significant increase in the number of deleterious pores. This increase may be partly related to the air-entraining effect of the excessive AE amount, as reported in previous studies [61]. Overall, the strength loss at high AE dosages may result from the combined effects of polymer agglomeration, reduced hydration, and air entrainment.

Figure 7e,f illustrate typical morphologies of AFt in the samples prepared without AE and with 5 wt.% AE, respectively. In the control sample, the needle-like AFt crystals were well-developed and densely interwoven. With the addition of 5 wt.% AE, the AFt needles became noticeably shorter and less abundant, and their crystal edges appeared blurred. This change is primarily due to the encapsulation effect of polymers, which hinders the growth of hydration products, resulting in the refinement of AFt crystals [62]. Furthermore, Figure 7f clearly revealed that extensive polymer films were distributed between and along the AFt crystals, interweaving with them to form an organic−inorganic network structure. The polymer films were observed to completely fill the gaps between AFt crystals, making pores barely visible. This microstructural attribute provides further insights into the mechanism by which AE improved the drying shrinkage of UHPC matrix. As the mechanical properties of organic polymer films were inferior to those of hydration products, the addition of AE evidently resulted in a reduction in the compressive strength of UHPC matrix. However, the formation of an organic−inorganic network composed of elastic AE films and hydration products, such as AFt, effectively improved the flexural strength of the composites.

### 2.8. Discussion

Based on the experimental results and microscopic characterization, the influence mechanisms of AE on the fresh and hardened properties of UHPC matrix can be summarised from two perspectives: (I) the retardation effect on early-age hydration and setting and (II) the improvement in volume stability and resistance to water penetration, as well as the promotion of flexural strength development, through the formation of polymer films. In both respects, the AE functions as an organic modifier that couples with the inorganic C-S-H gel skeleton of the matrix; the resulting material can thus be regarded as an organic–inorganic gel composite in which a polymer film network reinforces the host C-S-H gel phase.

**Figure 8:**
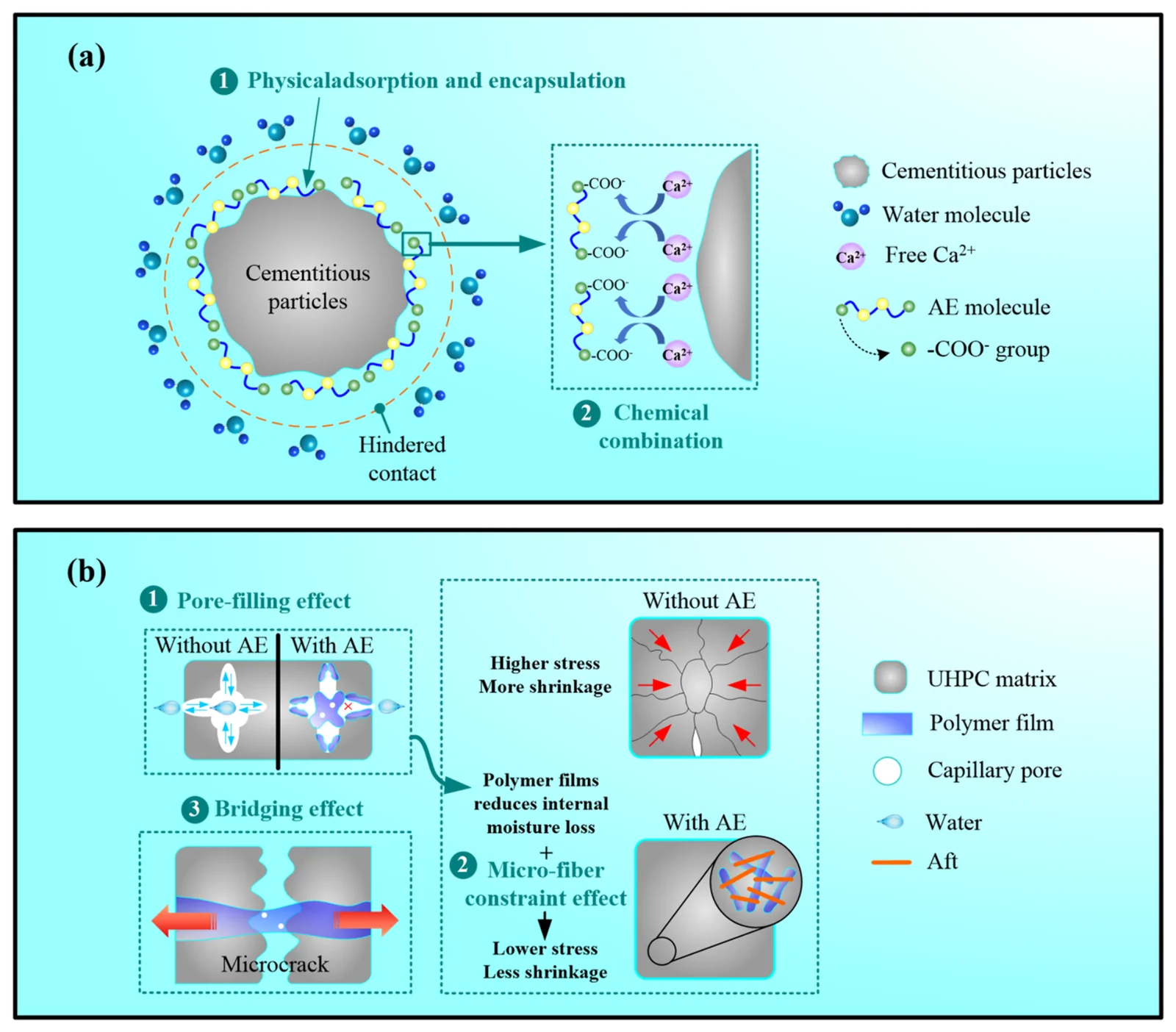
Schematic diagram of (**a**) the mechanism of AE’s influence on early hydration, and (**b**) the mechanism of AE’s improvement on UHPC matrix performance.

The addition of AE delays setting and early-age hydration, and this retarding effect becomes more pronounced with increasing AE content. As illustrated in Figure 8a, two primary mechanisms are responsible for this behavior. First, AE molecules are adsorbed onto the surface of cementitious particles, forming a physical barrier that impedes the direct contact between unhydrated clinker phases and water molecules. This encapsulation effect delays the onset of hydration reactions. Second, the abundant -COO functional groups on AE chains exhibit strong chelation with Ca^2+^ ions released during the early dissolution of cement, thereby reducing the free Ca^2+^ concentration in the pore solution. This complexation prolongs the induction period of C_3_S hydration and suppresses the nucleation and growth of hydration products [38]. Since C-S-H is the principal binding gel whose precipitation depends on the availability of free Ca^2+^ in the pore solution, this chelation retards the early formation of the C-S-H gel skeleton and reduces the volume of rigid gel phase available to carry load at later ages. Consequently, both the hydration heat evolution and the setting process are significantly retarded.

As curing progresses, AE gradually forms a continuous polymer film within the UHPC matrix, as confirmed by SEM observations (Figure 7). These films play a dual role in modifying the physical and mechanical properties of the hardened material (Figure 8b). These films provide pore-sealing and hydrophobic effects that slow the loss of interlayer and gel-pore water from the C-S-H gel, thereby alleviating the capillary tension that drives gel-skeleton contraction (consistent with the mechanism discussed in Section 2.4); this explains the lowered drying shrinkage observed at moderate AE dosages. Moreover, the interwoven arrangement of the polymer films with hydration products generates a “micro-fiber effect” that further constrains drying shrinkage, but only when the polymer is finely dispersed without agglomeration. The uniformly distributed polymer films also play a positive role in the development of flexural strength. The above effects are consistent with previous findings on AE-modified cementitious systems [63,64,65]. As a result, although the early-age flexural strength is slightly reduced due to retarded hydration, the 90-day flexural strength of the UHPC matrix modified with an appropriate amount of AE (5–7.5 wt.%) shows a certain improvement compared with the control group. In contrast, the addition of AE has a detrimental effect on compressive strength, particularly at higher dosages. This deterioration is attributed to two main factors. First, the elastic modulus of the polymer film is significantly lower than that of the hydration products, making the composite more compliant under compressive stress and introducing structural defects. Second, the encapsulation and chelation effects of AE reduce the overall degree of hydration, leading to a lower volume of C-S-H gel, the principal rigid binding phase. Nevertheless, at the optimal AE content of 5 wt.%, the negative impact on compressive strength is relatively limited, while the benefits in shrinkage reduction and water resistance are maximized. This balance is particularly relevant for large-volume UHPC applications, where the delayed hydration and reduced early heat release at 5 wt.% AE can help mitigate thermal stress and reduce the risk of thermal cracking. However, higher AE dosages may cause excessive retardation, compressive strength loss, polymer agglomeration, air entrainment, and increased harmful pores, which could adversely affect construction schedules and long-term durability. Therefore, the AE dosage should be carefully optimized, and large-scale field validation is recommended before practical application.

## 3. Conclusions

This work systematically investigates the effects of AE on the key properties of UHPC matrix, including setting time, mechanical performance, drying shrinkage, and capillary water absorption. The underlying mechanisms were analysed via hydration heat analysis, TG analysis and SEM. The main conclusions are summarised as follows:

(1) The addition of AE delays setting and early-age hydration, with the extent of this effect increasing as the AE dosage increases. This behavior helps to alleviate localised stress concentration caused by rapid early heat release in UHPC matrix, thereby reducing the likelihood of microcrack formation.
(2) The incorporation of AE reduces the compressive strength of the UHPC matrix, and this adverse effect becomes pronounced at high dosages. However, the addition of an appropriate amount of AE plays a certain promoting role in enhancing the flexural strength.
(3) UHPC matrix containing AE exhibits significant advantages in controlling drying shrinkage and capillary water absorption. These improvements are mainly due to the continuous polymer film formed within the matrix, which provides effective pore-sealing and hydrophobic effects, thus limiting internal moisture loss and hindering the intrusion of external solutions. Furthermore, the interwoven arrangement of the polymer film within the hydration products generates a “micro-fibre effect” that effectively constrains the development of drying shrinkage. The sample containing 5 wt.% AE demonstrates the best overall performance, with the 90-day drying shrinkage rate and capillary water absorption reduced by 25% and 27%, respectively, compared to the sample without AE.

Overall, the use of polymer emulsions offers a feasible approach to developing UHPC with low heat of hydration, suppressed shrinkage, and high resistance to water penetration, making it suitable for the construction of large-volume underwater engineering structures.

## 4. Materials and Methods

### 4.1. Raw Materials

**Figure 9:**
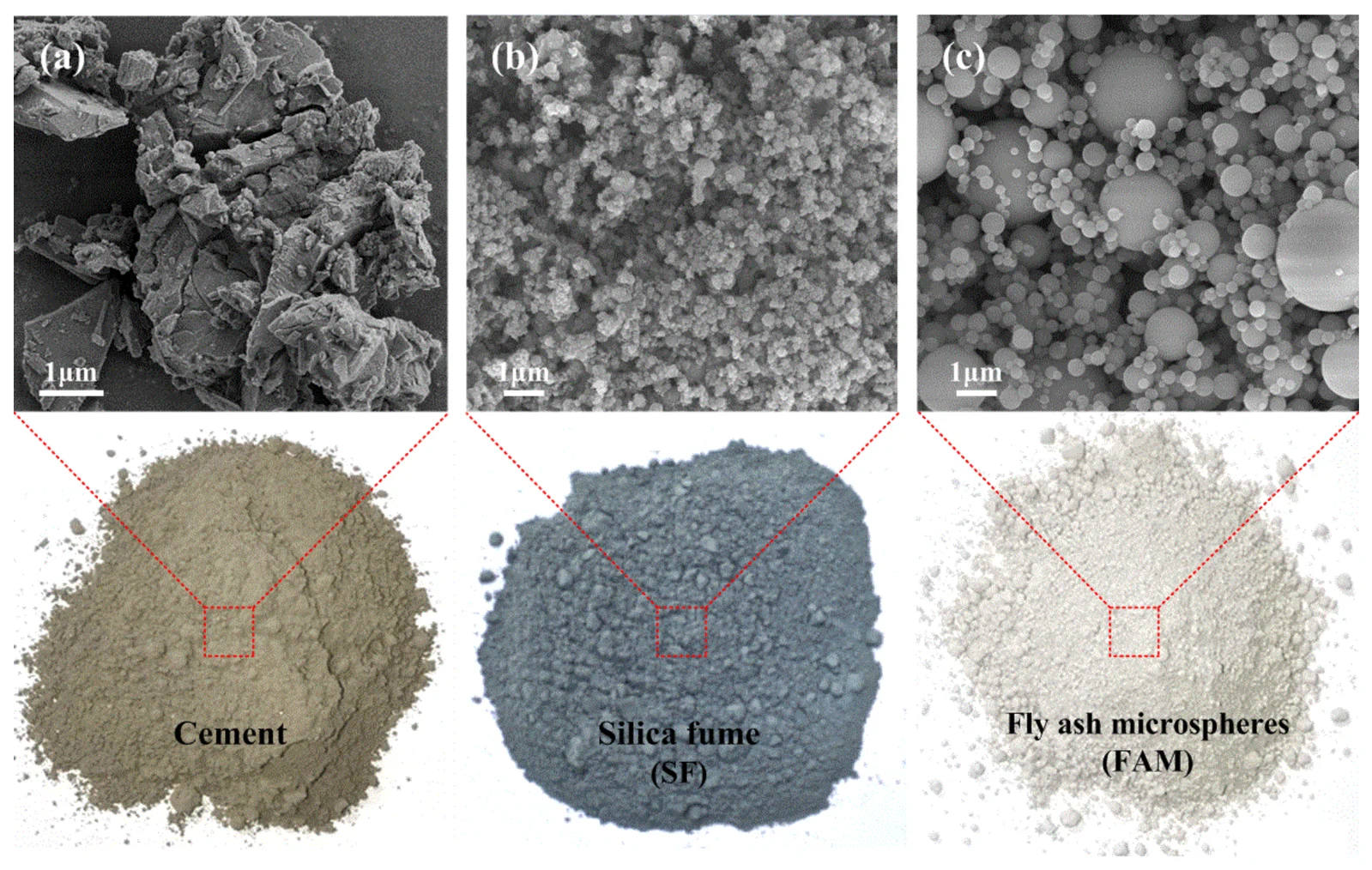
Macroscopic morphology and SEM images of cement (**a**), SF (**b**), and FAM (**c**).

The main cementitious material used in this study was a commercial P·O 52.5R Portland cement produced by Guangdong Yuebao Building Materials Co., Ltd. (Guangzhou, China). Silica fume (SF) and fly ash microspheres (FAM) were incorporated as supplementary cementitious materials and were supplied by Gansu Sanyuan Silicon Materials Co., Ltd. (Lanzhou, China) and Shijiazhuang Zhenyu New Material Technology Co., Ltd. (Shijiazhuang, China), respectively. Figure 9 shows the macroscopic morphology and SEM images of the cement, SF, and FAM particles.

**Figure 10:**
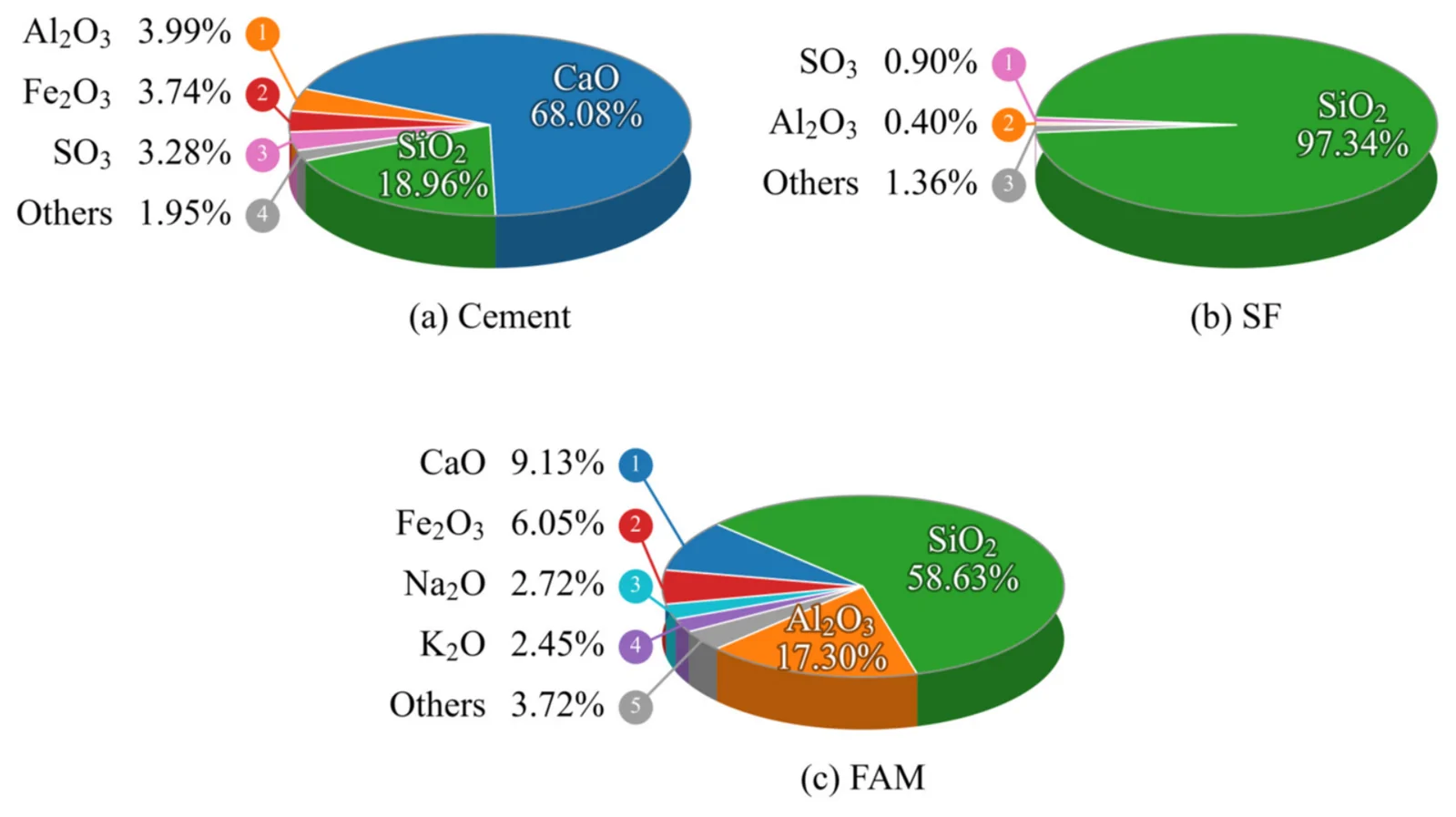
Chemical compositions of cement (**a**), SF (**b**), and FAM (**c**).

Furthermore, the detailed chemical compositions of the three cementitious materials were determined using an X-ray fluorescence spectroscopy (Rigaku ZSX Primus III+, Tokyo, Japan), and the results are shown in Figure 10. Cement was dominated by CaO (68.08%) and SiO_2_ (18.96%), with minor amounts of Al_2_O_3_ (3.99%), Fe_2_O_3_ (3.74%), and SO_3_ (3.28%), which is typical of ordinary Portland cement. SF consisted predominantly of SiO_2_, accounting for 97.34%. In contrast, FAM was primarily composed of SiO_2_ (58.63%) and Al_2_O_3_ (17.30%), along with appreciable amounts of CaO (9.13%) and Fe_2_O_3_ (6.05%). In particular, the combined content of SiO_2_, Al_2_O_3_, and Fe_2_O_3_ in FAM reached 81.98%, which is characteristic of a low-calcium, siliceous–aluminous fly ash.

**Figure 11:**
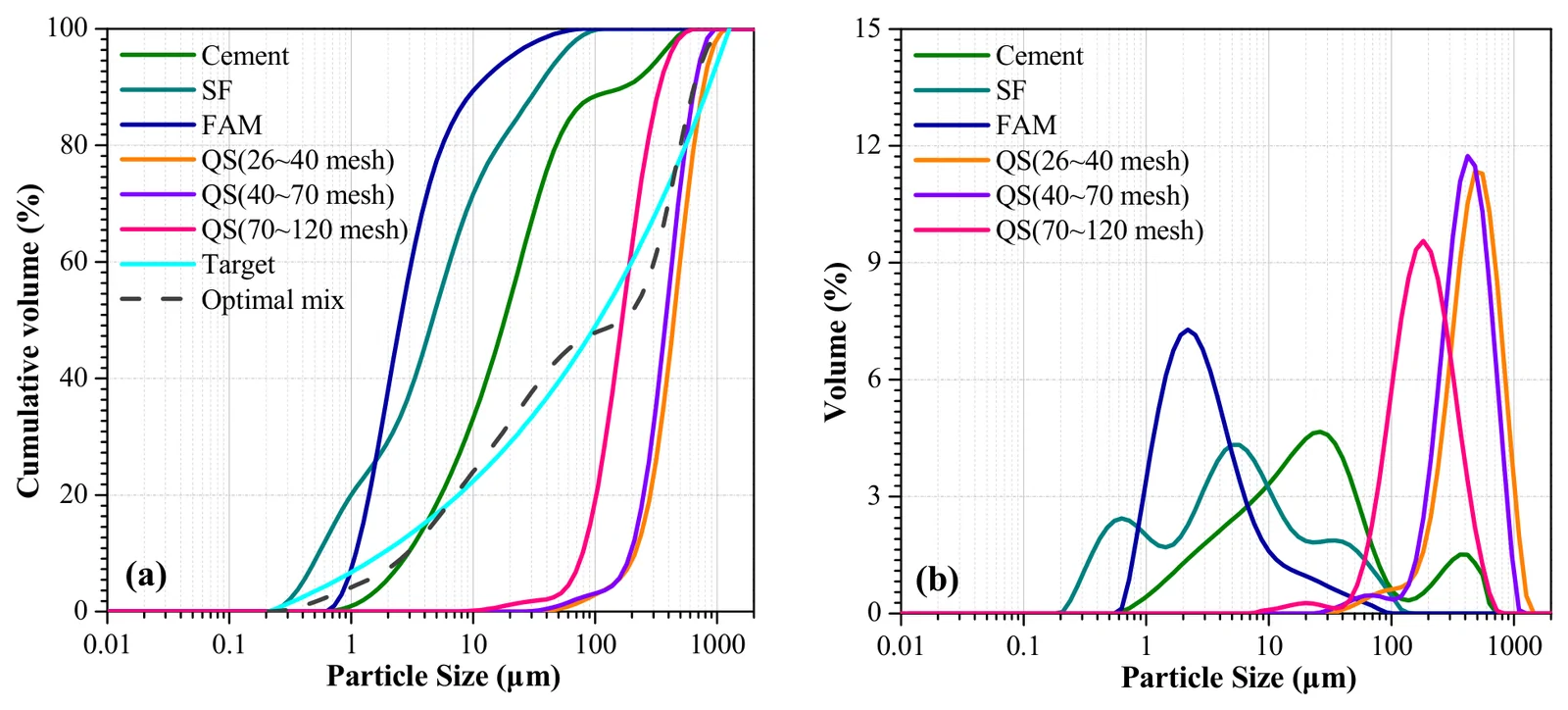
Particle size distribution of the raw materials: (**a**) cumulative volume distribution; (**b**) volume distribution.

Quartz sand (QS) was procured from Lidewang Co., Ltd. (Foshan, China). To ensure a well-graded aggregate system, three particle-size ranges were adopted, corresponding to 26–40 mesh (0.710–0.425 mm), 40–70 mesh (0.425–0.212 mm), and 70–120 mesh (0.212–0.125 mm). The particle size distributions of all raw materials were measured using a Mastersizer 2000 laser diffraction particle size analyser produced by Malvern Instruments Ltd. (Malvern, UK), and the corresponding results are presented in Figure 11. A modified Andreasen and Andersen particle packing model [66] was adopted to calculate the theoretical curve of packing density. On this basis, the optimal mixing ratios for all particles were calculated to maximise packing density, thereby forming a matrix with higher compactness.

**Figure 12:**
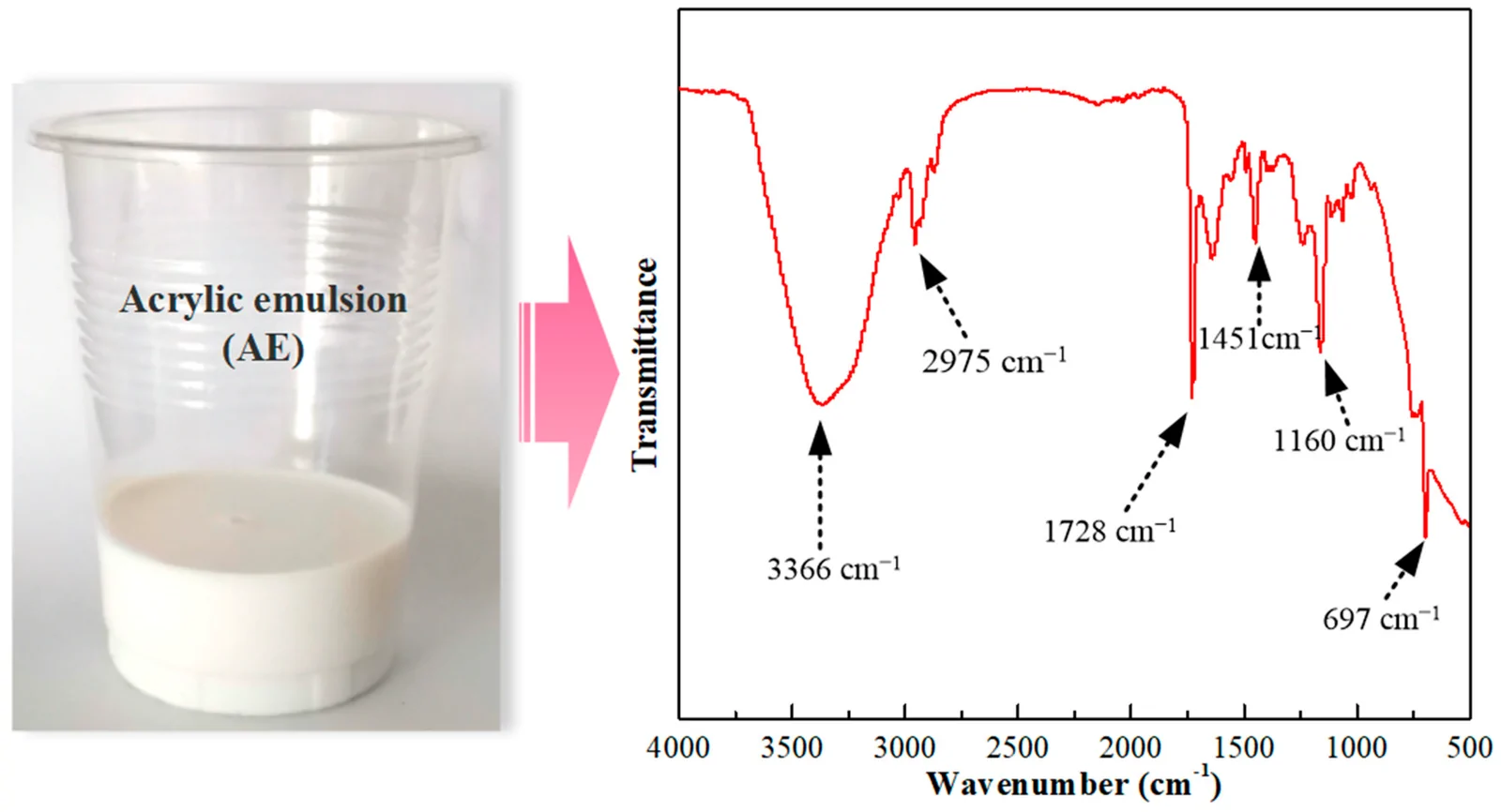
FTIR spectrum of AE.

The additive AE was supplied by Shenzhen Jitian Chemical Co., Ltd. (Shenzhen, China) with a solid content of 35% and a viscosity in the range of 1200–1600 cps. The physicochemical performance parameters of AE, as provided by the supplier, are listed in Table 1. The structure of AE was characterised by Fourier transform infrared (FTIR) spectroscopy using a Nicolet iS20 spectrometer (Thermo Fisher Scientific, Madison, WI, USA), and the results are shown in Figure 12. The FTIR spectrum of AE exhibited distinct characteristic peaks at 3366, 2957, 1728, 1451, 1160, and 697 cm^−1^. The broad band at 3366 cm^−1^ is attributed to the stretching vibration of the O–H groups in water. The peaks at 2957 cm^−1^ and 697 cm^−1^ originate from the stretching vibrations of –CH_3_ bonds in AE. A strong characteristic peak at 1728 cm^−1^ is ascribed to C=O stretching vibrations, indicating the presence of abundant COO^−^ groups on the surface of AE [67]. In addition, the characteristic absorption peak at 1160 cm^−1^ corresponds to the C–O–C stretching vibration of ester linkages in AE. To ensure good workability and compactness of the mixture, a polycarboxylate-based superplasticiser (SP) produced by Kezhijie New Materials Group (China) was used, featuring a water-reduction rate of 45% and a solid content of 35%.

### 4.2. Sample Preparation

In this study, UHPC matrix incorporating AE was prepared with four polymer-to-cement (P/C) weight ratios of 0, 5, 7.5, and 10 wt.% in order to systematically investigate the effects of AE addition on hydration behavior, mechanical properties, and drying shrinkage. For all mixtures, the water-to-cement ratio was kept at 0.18, while SF and FAM were introduced at a fixed dosage of 20 wt.% and 30 wt.% relative to the cement content, respectively. A polycarboxylate superplasticiser, equivalent to 5 wt.% of the cement, was further incorporated to ensure adequate fluidity of the mixture. The conventional UHPC matrix mix proportion adopted above is consistent with our previous studies [57].

**Figure 13:**
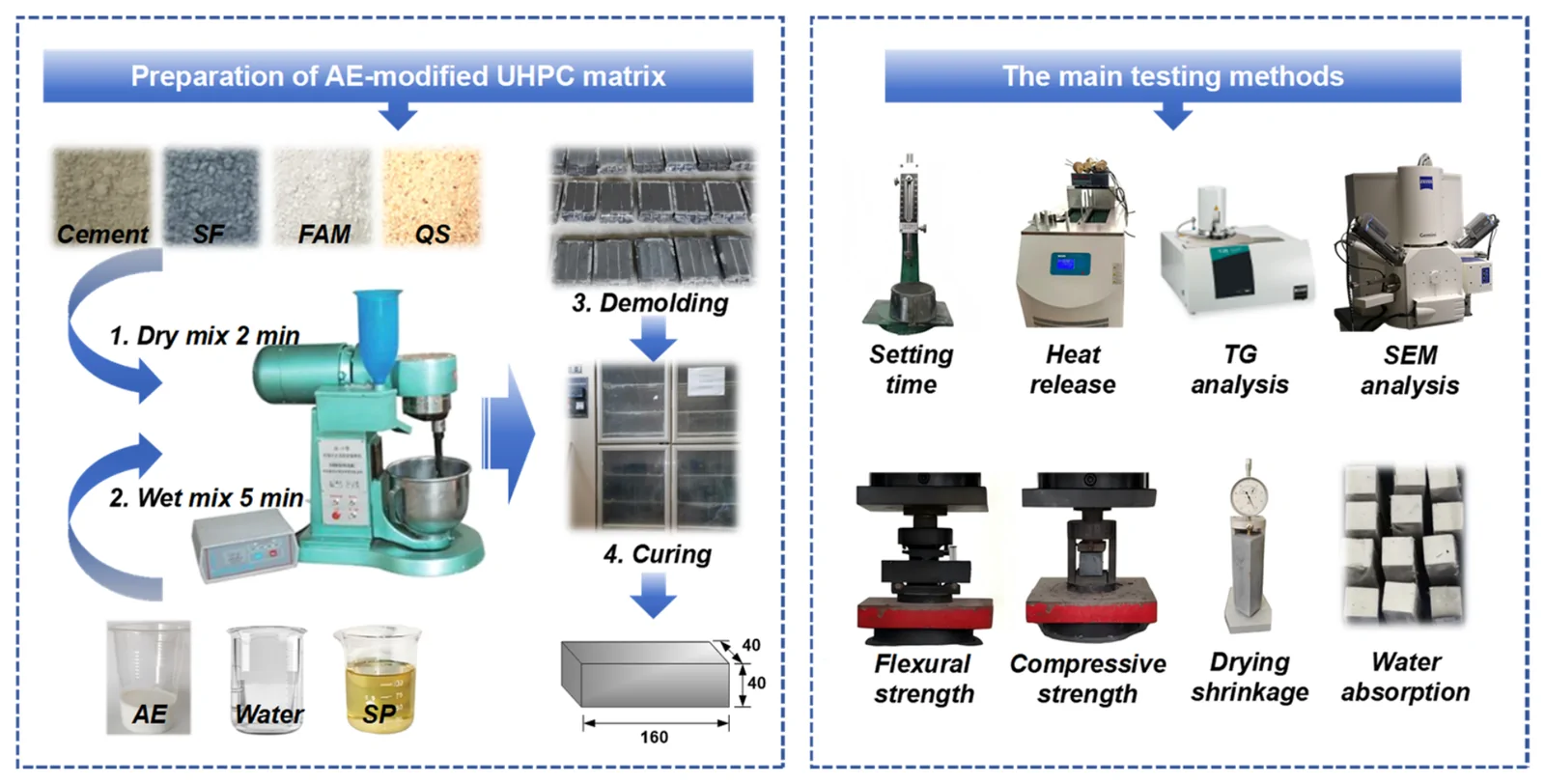
Preparation, curing, and testing of specimens.

The preparation steps for the AE-modified UHPC matrix were carried out as follows (as shown in Figure 13):

(1) The pre-weighed cement, SF, FAM, and QS were placed in a planetary mixer and dry mixing for 2 min;
(2) Subsequently, AE, mixing water, and the SP were gradually introduced into the dry mixture, followed by continuous wet mixing for 5 min to ensure uniform distribution of all components;
(3) After mixing, the fresh mixture was then immediately cast into triple moulds and manually compacted to eliminate entrapped air and improve densification;
(4) After 24 h, the specimens were demoulded and cured at 25 ± 5 °C under a relative humidity of 98 ± 2% until the required testing ages.

### 4.3. Test Methods

The setting time of the AE-modified UHPC paste (i.e., the matrix composition without quartz sand) was determined using the Vicat apparatus according to Chinese standard GB/T 1346-2011 [68], a method widely adopted in previous studies [69,70]. The heat release during hydration of the UHPC pastes (i.e., the paste composition without quartz sand) with varying AE contents was measured using a six-channel thermal activity monitor, with glass ampoules serving as the reaction vessels, following the procedure specified in Chinese Standard GB/T 12959-2008 [71]. All measurements were conducted at a constant ambient temperature of 20 °C, and the heat-flow signals of each mixture were automatically recorded by sensors for a total duration of 48 h (2 days). It should be noted that since the UHPC paste was externally mixed prior to testing, the heat flow generated during the initial hydration stage was considered inaccurate.

The flexural and compressive strengths of UHPC matrices with different AE contents were tested at curing ages of 3, 28, and 90 days on an MTS universal testing machine in accordance with Chinese standard GB/T 17671-2021 [72]. These curing ages were selected because 3 days reflects early-age strength and hydration, 28 days is the standard design age for cement-based materials, and 90 days represents long-term performance and the stabilized effect of polymer film formation. Flexural strength was measured using a three-point bending method at a loading rate of 50 N/s and a support span of 100 mm, using specimens of 160 mm × 40 mm × 40 mm. For each mix proportion, three specimens were prepared for three-point bending tests, and the average value of valid results was reported as the flexural strength. Subsequently, the broken specimens obtained after the bending tests were used for compressive strength measurements. The compressive loading area was 40 mm × 40 mm, and the loading rate was 2400 N/s. Six valid data points were obtained, and their average value was used as the representative compressive strength.

The drying shrinkage rate (*ε_t_*, μm/m) of the hardened UHPC matrix specimens was determined according to the Chinese standard JGJ/T 70-2009 [73]. It was calculated from the variation in specimen length at different curing ages (*t*, days), denoted as *L_t_* (mm), relative to the initial length measured after demoulding (*L*_0_, mm), using the following formula:

(1) $$\varepsilon_{t}=\frac{L_{0}-L_{t}}{L_{0}}$$

Capillary water absorption of the AE-modified UHPC matrix was evaluated following the German standard DIN 52617 [74]. Prior to testing, all specimens were oven-dried at 60 °C for approximately 7 days to remove residual moisture. After cooling to room temperature, the non-permeable surfaces were coated with paraffin wax, and the initial weights of each specimen were recorded. The samples were then placed in clean water for testing, with the water level maintained about 1 cm above the bottom surface of the specimens.

To investigate the effects of different AE dosages on the formation of hydration products in UHPC matrix, powdered samples obtained by grinding and sieving through a 75 μm sieve were heated from 40 °C to 1000 °C at a heating rate of 10 °C/min using a TGA55 thermal analyzer (TA Instruments, New Castle, DE, USA) under nitrogen atmosphere. Subsequently, the mass losses of corresponding components were determined from the recorded TG and derivative TG (DTG) curves. Furthermore, the microstructural features of UHPC matrix with different AE contents were examined by SEM using a MIRA LMS instrument (TESCAN, Czech Republic) operated at an accelerating voltage of 15 kV. Prior to all measurements, the samples were immersed in anhydrous ethanol to stop hydration reactions.

## Figures and Tables

**Table 1:** Physicochemical characteristics of AE.

Viscosity (cps)	pH	Solid Content (%)	Glass Transition Temperature (Tg, ℃)
1200–1600	6–7	35	23

## Data Availability

The original contributions presented in this study are included in the article. Further inquiries can be directed to the corresponding author.
